# The balance between apoptosis and autophagy regulates testis regression and recrudescence in the seasonal-breeding South American plains vizcacha, *Lagostomus maximus*

**DOI:** 10.1371/journal.pone.0191126

**Published:** 2018-01-31

**Authors:** Candela R. González, María L. Muscarsel Isla, Alfredo D. Vitullo

**Affiliations:** 1 Centro de Estudios Biomédicos, Biotecnológicos, Ambientales y Diagnóstico- Universidad Maimónides, Buenos Aires, Argentina; 2 Consejo Nacional de Investigaciones Científicas y Técnicas, CONICET, Buenos Aires, Argentina; University of Hyderabad, INDIA

## Abstract

Mammalian testis undergoes deep changes in their architecture and function during photoregression conditions in seasonal breeders. Particularly, the testicular mechanisms that regulate the transition between the active (functional) and inactive (regression) stage vary between species. The aim of the present study was to analyze the incidence of proliferation, apoptosis and autophagy in the testicular seminiferous ephitelium of a seasonal breeder, *Lagostomus maximus*, during the annual reproductive cycle. We observed that proliferating spermatogonia increased from the active testis until reaching the maximum peak in the activating testis. During the annual reproductive cycle, the quantity of apoptotic-TUNEL positive spermatogonia and meiotic germ cells was constant and this might be regulated by the members of the BCL2 family. Only in the activating testis, apoptosis of germ cells was almost undetectable. The analysis of the autophagic-related proteins BECN1 and LC3 showed their localization in Leydig cells and the germ cells in the active and activating testis. In the inactive testis, BECN1 and LC3 ceased to be immunolocalized within the seminiferous tubules and the mRNA expression of both regulators decreased. Moreover, the expression of *BECN1* and *LC3* and also the apoptotic index were up regulated in testicular cultures subjected to nutritional stress. These results suggest a possible interaction between apoptosis and autophagy in the active and activating testis (characterized by high metabolic requirement and nutrient), where autophagy could promote survival over cell death. In the inactive testis, the absence of autophagic-related proteins might explain the massive loss of germ cells, suggesting that autophagy plays new and key role in the alterations of the seminiferous epithelium during photoregression.

## Introduction

In non-seasonal breeding mammals, the balance between apoptosis and proliferation maintains spermatogenic activity throughout the reproductive lifespan. However, seasonal-breeding animals show a particular case of spermatogenesis regulation since their testes annually undergo seasonal cycles of activation and inactivation [1–3]. During the transition of the breeding to the non-breeding stage, changes in reproductive function are modulated by the photic stimuli and the testes undergo a drastic reduction in weight and mass. A combination of substantial reduction in cellular size, decreased cell division rate and massive germ cell depletion, turn the seminiferous tubules into cords containing mainly spermatogonia and Sertoli cells, comparable to an immature testicular stage [1, 4–6].

It has been proposed that during testicular regression and recrudescence the alteration of the seminiferous epithelium is related to changes in the apoptotic process [2] and this has been widely reported in the Syrian hamster [7], the white-footed mouse [8,9] and the European brown hare [10]. In this context, different studies suggest that both the intrinsic and extrinsic pathways of apoptosis are implicated [2,11]. For instance, an increase in *Fas* expression in spermatogenic cells after exposure to short photoperiod has been reported in mice [8] and the participation of Fas, Bcl-xL, Bax, and p53 are thought to be involved in germ cell apoptosis induction after short photoperiod exposure in the Syrian hamster [12]. Recent studies have proposed that apoptosis or proliferation is not the cause of testicular regression [3,13]. Thus, the data so far described point out that the processes that regulate testicular regression are not uniform among species and pinpoints the importance of incorporating the study of new animal models.

In the last years, autophagy has gained relevance as a mechanism that, acting in concert with the intrinsic pathway of apoptosis, controls tissue homeostasis and cell survival [14,15]. Autophagy can be triggered by different causes of environmental stress such us nutritional deficiencies, hypoxia or exposure to high temperatures [15–17]. This process involves the degradation of cellular components through lysosomal machinery and is thought to be required for normal turnover of cellular components, especially in response to starvation [14,15]. The interaction between apoptosis and autophagy is complicated and depends on the cellular context. Autophagy can act both as an alternative death pathway to apoptosis and as stress adaptation mechanism to avoid cell death [15,18]. Nevertheless, the information regarding the role of autophagy in the testis is scarce and has not been further studied in testicular regression yet.

Aimed to increase our understanding on the role of autophagy and apoptosis in male germ line during the active and inactivating stages of the testis, we evaluated an emerging seasonal breeding rodent model, the South American plains vizcacha (*Lagostomus maximus*). Males of *L*. *maximus* are sexually active during the long days of summer and completely inactive in the winter where spermatogenesis is interrupted. In between, they undergo two transitional periods in autumn and springtime [19–21]. However, reproduction in the adult male vizcacha is yet not well understood and the mechanisms beyond testicular regression remain unknown. Here, we report a possible interaction between the autophagy and apoptosis processes that during the active and activating reproductive stages of the testis, autophagy could promote survival over cell death. We propose that the massive loss of germ cells during testicular regression might be related to the absence of the expression of autophagic-related proteins. To our knowledge, this is the first study showing the contribution of autophagy to the testicular regression.

## Materials and methods

### Animals and tissue collection

Plain vizcachas, *Lagostomus maximus*, were trapped from a resident natural population at the Estación de Cría de Animales Silvestres (ECAS), Ministry of Agriculture, Villa Elisa, Buenos Aires Province, Argentina, using live-traps located at the entrance of burrows. The protocol of this study was reviewed and approved by the Institutional Committee on the Care and Use of Experimental Animals (CICUAE-Universidad Maimónides). The number of animals captured was approved by the Ministry of Agriculture Authority of the Buenos Aires Province Government. Handling and euthanasia of captured animals were performed in accordance with the standards defined by the Guide for the Care and Use of Laboratory Animals (CCAC 2002) and Guidelines on the Care and Use of Wildlife (CCAC 2003) from the Canadian Council of Animal Care.

Animals were anesthetized with xylazine/ketamine (1:9), bled by intracardiac puncture and immediately euthanized by administration of 0.2 ml/ kg body weight Euthanyl (sodium pentobarbital, sodium diphenyl hydantoinate; Brouwer, Buenos Aires, Argentina) by trained technical staff. Testes were removed and immediately placed in 4% paraformaldehyde, kept at –70°C for molecular analysis or used for tissue culture studies. The testes were collected from 37 males captured during the main breeding season, which extends from January to March (Active, n = 11), during the quiescence period in July and August (Inactive, n = 11) and during two transitional periods in May (Inactivating, n = 7) and September (Activating, n = 8). For tissue culture studies, the testes of 6 active adult animals were used. Reproductive status was assessed on the basis of capture time, body and testis weight and testicular histology (Table 1).

**Table 1:** Males vizcachas analyzed in this study.

Stage	Period of capture	Testis weight (g)	Body weight (Kg)	Presence of late meiotic stages	Individuals analyzed
Active	January-March	4.78 ± 0.48^a^	6.28 ± 1.48^a^	Yes	11
Inactivating	May	3.91 ± 0.21^a^	5.64 ± 1.12^a,c^	Scarse	11
Inactive	July-August	1.83 ± 0.16^b^	3.81 ± 0.68^b^	No	7
Activating	September	2.82 ± 0.23^a,b^	4.32 ± 0.96^c^	Scarse	8

Values indicate mean ± SEM. Different letters indicate significant differences between groups (p<0.05).

### Testicular histology and morphometry

Fixed testes were embedded in paraffin and serially cut into 5-μm-thick sections, mounted onto cleaned coated-slides, dewaxed in xylenes, rehydrated in decreasing graded alcohols, washed in tap water, and processed for routine haematoxylin–eosin staining. For each specimen, at least 3 to 5 slides were stained for general histology inspection. The volumetric proportions of the testicular tissue components were determined by light microscopy using a 441-intersection grid placed in the 10X ocular of a light microscope [22,23]. Briefly, 15 fields chosen randomly (6615 points) were scored for each animal at a 400X magnification. Points were classified as one of the following: seminiferous cords, comprising tunica propia, epithelium and lumen; Leydig cells; blood vessels and lymphatic spaces and connective tissue. The results of the testicular proportions were expressed as percentages.

### Immunohistochemistry

Mounted paraffin sections were dewaxed in xylene, rehydrated in decreasing graded alcohols and washed in tap water. Endogenous peroxidase activity was inhibited in tissue sections using 0.5% v/v H2O2/methanol for 20 min at room temperature. Then, sections were blocked for 1 h with 15% normal goat serum in phosphate buffered saline (PBS) and then incubated overnight at 4°C with the primary antibody rabbit anti-PCNA (1:200 diluted ab2426, Abcam, UK); rabbit anti-BAX (1:200 diluted P-19, sc-526, Santa Cruz Biotechnologies, USA); rabbit anti-BCL2 (1:100 diluted ab7973, Abcam, UK); rabbit anti-CASPASE 3 (1:300 diluted AF835, RyD Systems, USA); rabbit anti-BECLIN 1 (1:500 diluted ab62472, Abcam, UK) or rabbit anti-LC3B (1:200 diluted ab48394, Abcam, UK). The, sections were rinsed in PBS and incubated for 1 h at room temperature with the appropriate 1:200 diluted biotinylated secondary antibody (Vector Labs, Peterborough, UK). After further washing in PBS, sections were incubated for 30 min with 1:100 diluted streptavidin-peroxidase complex (ABC kit, Vector Labs, Peterborough, UK). Finally, development of peroxidase activity was performed with 0.05% w/v 3,3’-diaminobenzidine and 0.1% v/v H2O2 in Tris-HCl. Negative controls were processed simultaneously by omitting the primary antibodies or pre-absorbing the primary antibody with specific synthetic peptides.

### TUNEL assay

Apoptosis-dependent DNA fragmentation was detected in paraffin-embedded sections by terminal deoxynucleotidyl transferase-mediated deoxyuridine triphosphate nick end labelling (TUNEL) technique, using the *In Situ* Cell Death Detection Kit (Roche Diagnostics GmbH, Germany or Basel, Switzerland) fluorescein-tagged nucleotides, according to the manufacturer’s protocol (AP Protocol, No. 11684809910). Treated sections were examined in an Olympus BX40 microscope with conventional epifluorescence with ultraviolet illumination. In order to ascertain negative results, TUNEL-processed sections were incubated with 10 IU/ml DNase II (Sigma) in 50 mM Tris–HCl, pH 7.5, 10 mM MgCl2, and 1 mg/ml BSA for 10 min at room temperature. The, slides were treated according to the TUNEL protocol. Images were captured with an Olympus Camedia C-5060 camera.

### Quantification of proliferating and apoptotic germ cells

The quantification of positive spermatogonia for PCNA or TUNEL was performed by counting all positive and negative cells for each marker by seminiferous tubule, in at least 30 seminiferous tubules randomly-chosen in a total of 3 sections per animal [3,13]. In the case of TUNEL, primary and secondary spermatocytes and early and late spermatids were also counted. Quantification of the positive cells was expressed as percentages for each specific cell type. PCNA and TUNEL reactive cells were counted in immunohistochemistry-treated sections using an Olympus BX40 microscope (Tokyo, Japan) at 1000× magnification. Counting was performed independently by two observers. Cells were recognized according to nuclear and cytoplasmic characteristics [24–26].

### RNA isolation and real time PCR

Total testicular RNA was extracted with TRIzol (Invitrogen) according to the manufacturer’s instructions. Total RNA (3 μg) was treated with DNaseI (Invitrogen) and used for the reverse transcription reaction in a 20 μl reaction containing M-MLV reverse transcriptase (200 U/μl, Promega, Madison, WI, USA) and random hexamer primers (Biodynamics, Buenos Aires, Argentina). Reverse-transcribed cDNA was used for quantitative polymerase chain reaction (PCR) using SYBR Green PCR Master Mix and specific forward (F) and reverse (R) primers (Table 2) in a Stratagene MPX500 cycler (Stratagene, La Jolla, CA, USA). Primers were used at a concentration of 0.3μM in each reaction. The cycling conditions were as follows: step 1, 10min at 95°C; step 2, 30sec at 95°C; step 3, 30sec at 55°C; step 4, 30sec at 60°C; repeating steps 2 to 4 forty times. Data from the reaction were collected and analyzed by the complementary computer software (MxPro3005P v4.10 Build 389, Schema 85, Stratagene, USA). Melting curves were run to confirm specificity of the signal. Relative quantitation of gene expression was calculated using standard curves and normalized to *Gapdh* each sample. Quantitative differences in cDNA target between samples were assessed by the mathematical model of Pfaffl [27]. An expression ratio was determined for each sample by calculating (Etarget)ΔCt(target)/ (EGAPDH)ΔCt(GAPDH), where E is the efficiency of the primer set and ΔCt = Ct (normalization cDNA)—Ct (experimental cDNA). The amplification efficiency of each primer set was calculated from the slope of a standard amplification curve of log (ng cDNA) per reaction vs. Ct value (E = 10-(1/slope)). Efficiencies of 2 ± 0.1 were considered optimal.

**Table 2:** Oligonucleotide primers used for real time PCR amplification of cDNA obtained after reverse transcription from testes of *Lagostomus maximus*.

Target (accesion number)	Sequence of primer 5’-3’	TM (C)	Amplified product (bp)
*BAX* (NM_138761.3)	F:GCATCGGGGACGAACTGG R:GTCCCAAAGTAGGAGAGGA	60	307
*BCL2* (NM_000633.2)	F:GCCTTCTTTGAGTTCGG R:GGGTGATGCAAGCTCC	60	250
*BECN1* (NM_053739.2)	F:TTCAAGATCCTGGACCGAGTGAC R:AGACACCATCCTGGCGAGTTTC	60	142
*LC3* (NM_022867.2)	F:CATGCCGTCCGAGAAGACCT R:GATGAGCCGGACATCTTCCACT	60	69
*GAPDH* (NM_008084)	F:CCAGAACATCATCCCTGCAT R:GTTCAGCTCGGGATGACCTT	60	67

F: forward, R: reverse, TM: temperature of melting, bp: base pairs.

### Western blot

Testes were dissected in fragments and 3 ml of lysis solution were added per gram of tissue. The lysis solution contained RIPA buffer (1% Igepal, 0.5% sodium deoxycholate, 0.1% sodium dodecyl sulfate) with protease inhibitors (200 mM Phenylmethylsulfonyl fluoride, 100 mM sodium orthovanadate, 10 μM Leupeptin, 1 μM Pepstatin and 10 μM Aprotinin (all reagents from Sigma, Saint Louis, MO, USA). Samples were homogenized with a high-speed homogenizer and centrifuged at 10,000 rpm for 10 min at 4°C. Protein content was determined with Bradford assay (BioRad). Total protein (15μg) were separated by one-dimensional SDS-PAGE (12%) and then transferred onto PVDF membranes (Immobilon-P Transfer membrane, Millipore, Bedford, USA). Membranes were blocked for 1 h in PBS with 5% non-fat dry milk and incubated with rabbit anti-LC3 antibody (1:1000, Sigma, Saint Louis, Missouri, USA). Then, samples were incubated for 2 h with goat anti-rabbit IgG (H+L) horseradish peroxidase conjugated secondary antibody (1:3000, GE, Amersham, Fairfield, Connecticut, USA). The immunoreactive product was visualized using the enhanced chemiluminescence system (ECL plus or prime GE, Amersham, Fairfield, Connecticut, USA). Densitometry was performed on Scion Image for Windows software (Scion Corporation 2000–2001). LC3-II expression was normalized to β-actin (Sigma, Saint Louis, Missouri, USA).

### Tissue culture

Testes of adult animals (n = 6) were decapsulated and dissected with a scalpel in 32 equivalent fragments of approximately 0.5 x 0.5 cm under sterile conditions. Two fragments per well were placed in 2 ml of DMEM media (Invitrogen) supplemented with 10% fetal bovine serum (FBS) and 1% penicillin/streptomycin (p/s) (*rich medium*) or in DME media (Invitrogen) supplemented with 1% p/s (*starvation medium*) [28,29]. Testis fragments were incubated at 37°C in humidified atmosphere with 5% CO_2_ for 3, 6 and 12 h [28,29]. Finally, they were washed and frozen at -80°C for molecular biology studies.

### Statistical analysis

Mean and standard error (SEM) were calculated and the GraphPad Prism Software (version 5.0 for Windows, GraphPad Software, San Diego, CA, USA) was used for one-way analysis of variance. The Bonferroni test was used when differences between more than two groups were compared. Differences between two groups were analyzed with Student’s *t*-Test. In both cases, differences were considered statistically significant if *p*<0.05.

## Results

### The size of the seminiferous tubules decreased during the non-breeding season

**Fig 1:**
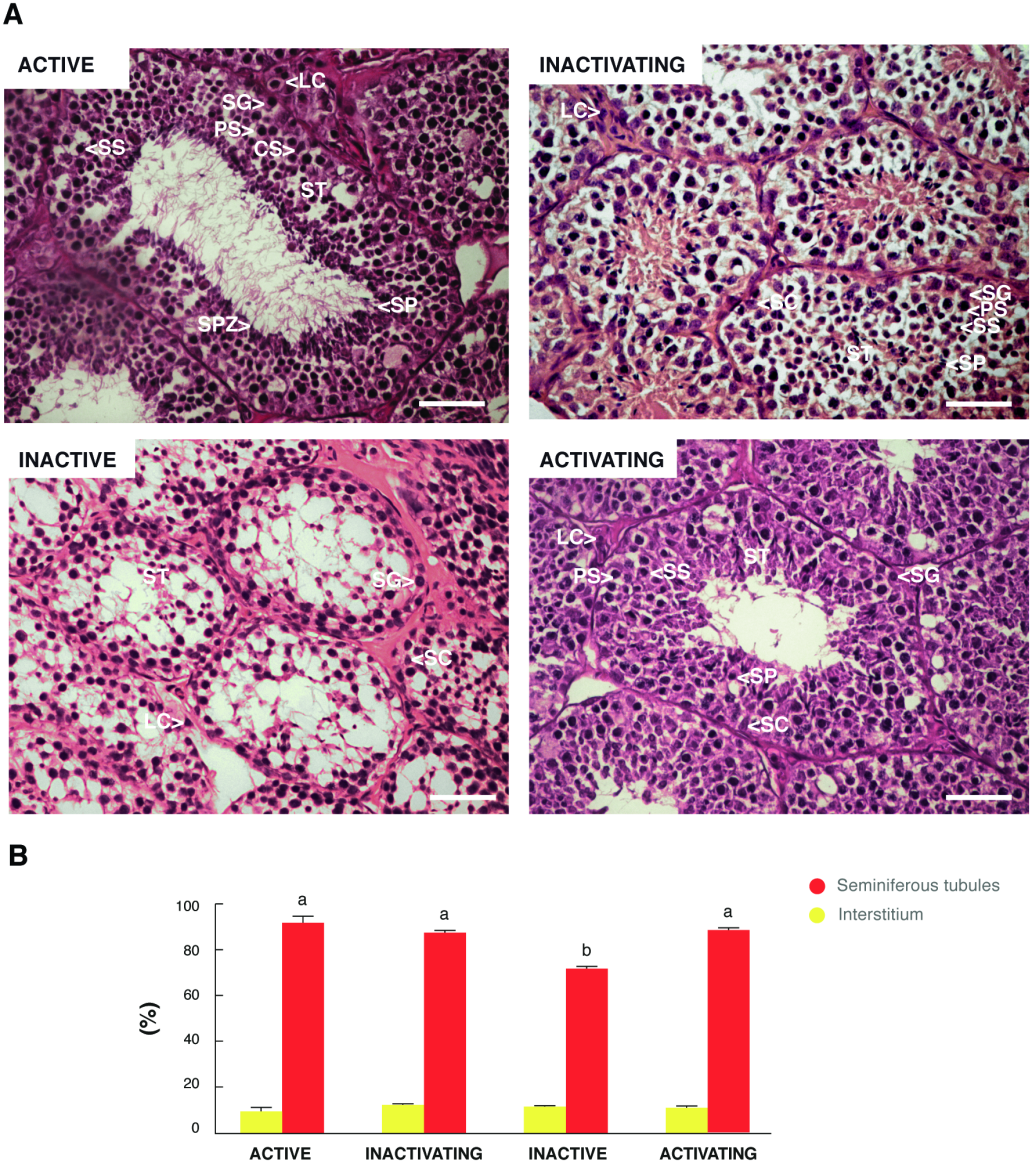
General histology and testicular morphometry of *L*. *maximus* during the annual reproductive cycle. Testicular histology (A) and volumetric proportions of the tubular and interstitial compartments (B) of the adult active, inactivating, inactive and activating testis. ST: seminiferous tubules, SG: spermatogonia; PS: primary spermatocyte; SS: secondary spermatocyte; SP: spermatid; SPZ: spermatozoid; SC: Sertoli cell; LC: Leydig cell. Scale bar: 50 μm. Values in (B) indicate mean ± SEM. Different letters indicate significant differences between groups for each testicular compartment (*p*<0.05).

In the adult active testis, the seminiferous tubules were large in diameter and spermatozoa were present in the lumen (Fig 1A). In the inactive testis, seminiferous tubules were reduced in size and in most cases, turned into cords, composed mainly of spermatogonia and Sertoli cells (Fig 1A). The inactivating and activating testis showed intermediate histological characteristics between the active and inactive stages. The inactivating testis presented a slight decrease in the size of the seminiferous tubules with signs of early testicular regression, while activating testis showed some seminiferous tubules with spermatogenesis and histological signs of gonadal recovery (Fig 1A). Morphometric studies revealed that the tubular compartment decreased significantly in the inactive testis respect to the other groups (Fig 1B). The volumetric proportions of the interstitial compartment did not show significant variations during the course of the annual reproductive cycle (Fig 1B).

### Proliferation increased during testicular regression

**Fig 2:**
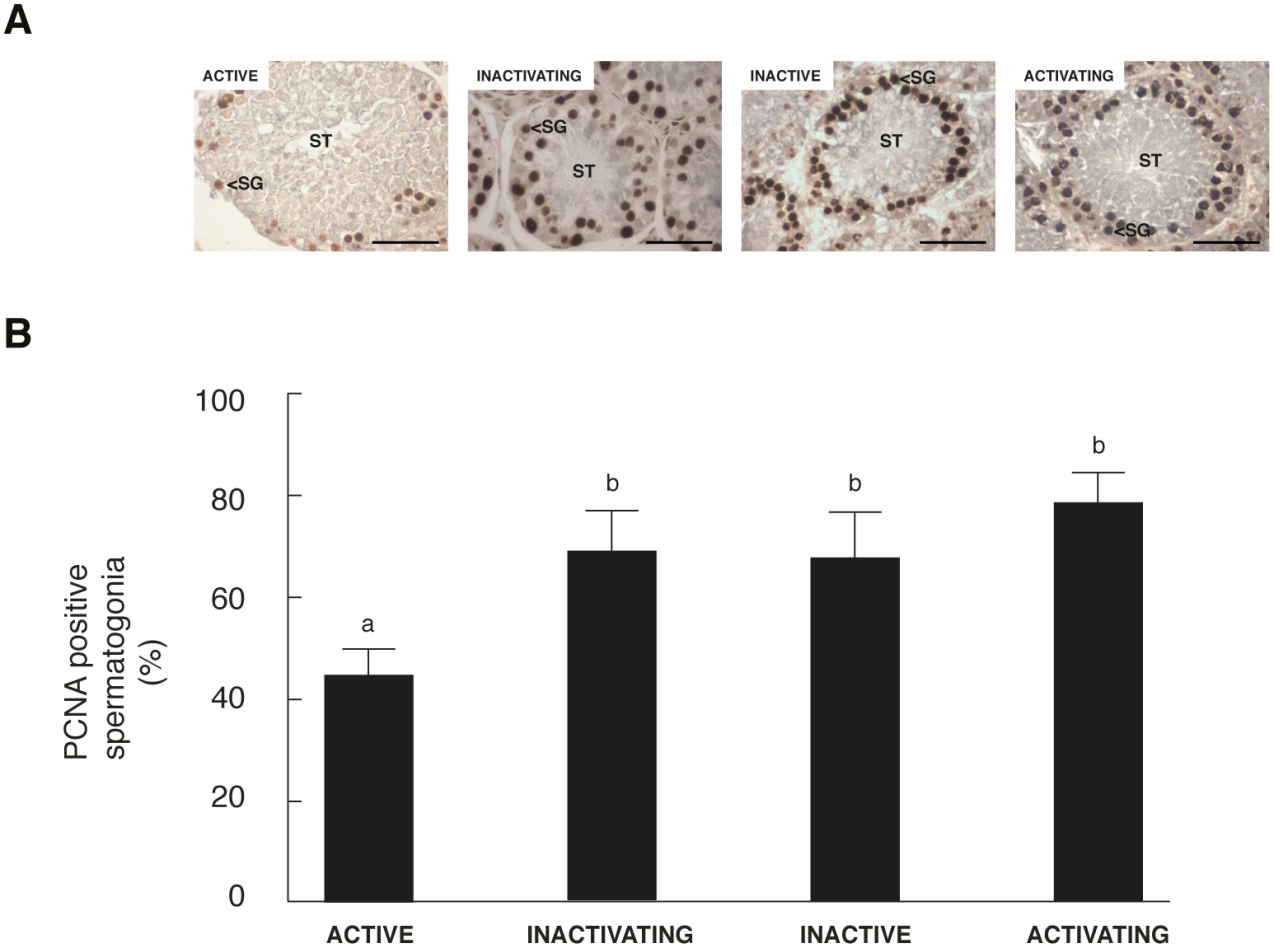
PCNA positive spermatogonia during the annual reproductive cycle of *L*. *maximus*. Immunolocalization of PCNA (A) and quantification of immunohistochemistry assay represented as the percentage of PCNA-immunoreactive spermatogonia (B) in adult active, inactivating, inactive and activating testis. ST: seminiferous tubules, SG: spermatogonia. Scale bar: 50 μm. Values in (B) are expressed as mean ± SEM. Different letters indicate significant differences between groups (*p*<0.05).

PCNA is expressed during late G1/S phase of the cell cycle and it has been used extensively in the identification of proliferating spermatogonia. Here, the quantification analysis showed that the proportion of proliferative spermatogonia increased as the annual cycle progressed being significantly higher in the inactivating, inactive and activating testis respect to active testis (Fig 2).

### Apoptosis is suppressed during the activating stage of the reproductive cycle

**Fig 3:**
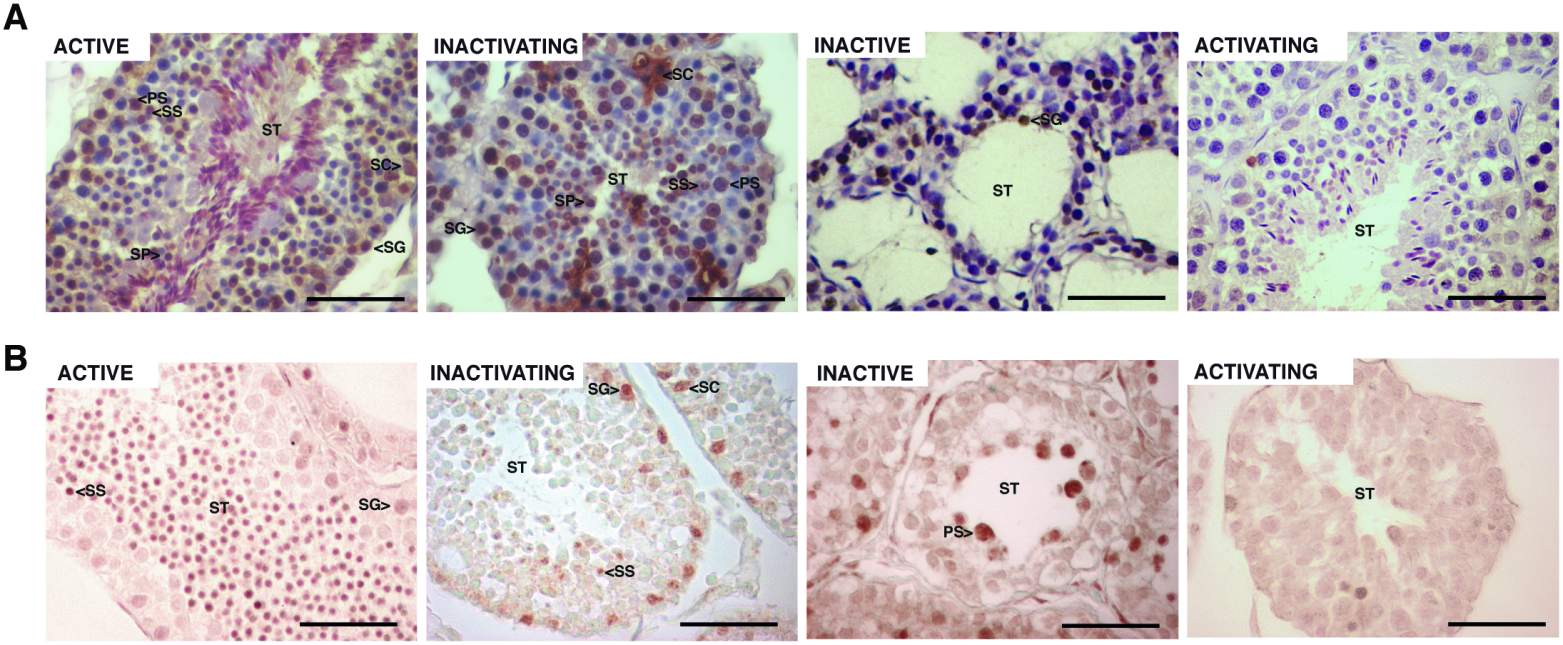
Germ cell apoptosis during the annual reproductive cycle of *L*. *maximus*. TUNEL assay (A) and immunohistochemical staining for CASP3A (B) in adult active, inactivating, inactive and activating testis. ST: seminiferous tubules, SG: spermatogonia; PS: primary spermatocyte; SS: secondary spermatocyte; SP: spermatid; SC: Sertoli cell. Scale bar: 50 μm.

TUNEL assay showed a stage-specific pattern during the annual reproductive cycle. Active, inactivating and inactive testes showed high rates of apoptotic germ cells at different stages of the spermatogenic cycle (Fig 3A). The activating testis presented isolated positive germ cells for TUNEL, indicating scarce apoptosis (Fig 3A). Some apoptotic Sertoli cells were detected in active and inactivating testis (Fig 3A). In line with TUNEL assay, CASP3A immunoreactive cells were abundant in the active, inactivating and inactive testis and very rare or undetectable in the activating testis (Fig 3B). Table 2 shows the quantification of TUNEL-positive germ cells at different stages of the cycle. The percentage of apoptotic spermatogonia per seminiferous tubule did not vary during active, inactivating and inactive stages, except that in activating stage the value was less than 1%. Similarly, primary, secondary and early spermatids percentages were also not varying in active and inactivating stages while could not be detected in inactive stage and remained less than 1% in activating stage (Table 3). Apoptotic late spermatids were almost undetectable in the active testis and significantly lower respect to the inactivating testis (Table 3).

**Table 3:** TUNEL-positive germ cells during the annual reproductive cycle of *Lagostomus maximus*.

Stage	SG (g)	PS (%)	SS&SP_E_ (%)	SP_L_ (%)
Active	24.68 ± 9.03	38.30 ± 2.39	27.59 ± 6.78	1 ± 0.87
Inactivating	25.58 ± 5.50	47.16 ± 10.46	35.28 ± 6.23	9.09 ± 0.89*
Inactive	19.88 ± 6.06	ND	ND	ND
Activating	<1	<1	<1	<1

SG: Spermatogonia; PS: Primary spermatocyte; SS: Secondary spermatocyte; SP_E_: Early spermatid; SP_L_: Late spermatid; ND: Not detectable stage. Values indicate mean ± SEM.

*p<0.05

### BCL2 gene family expression in testicular regression

**Fig 4:**
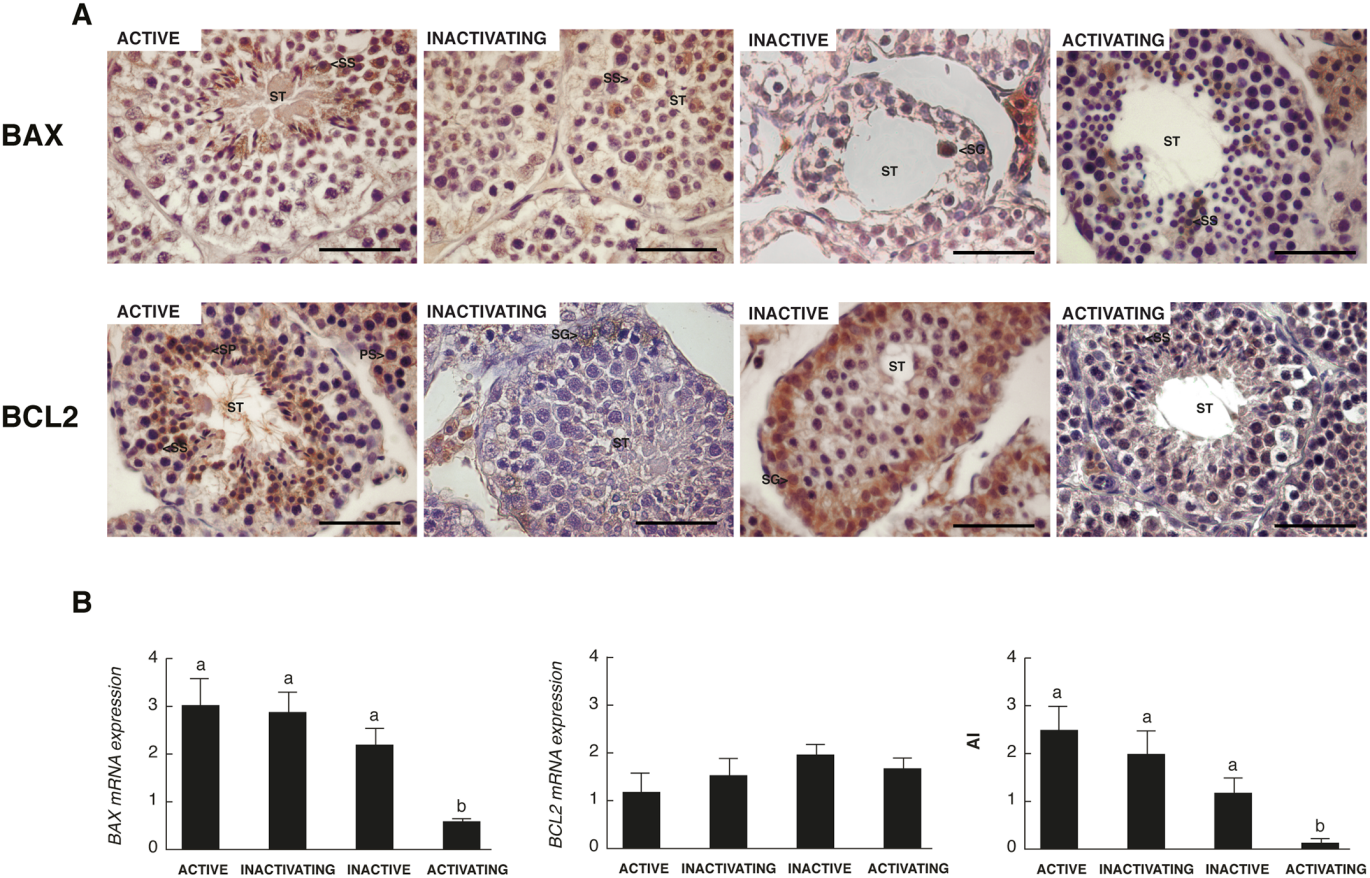
Expression of the BCL2 family members in the testis of *L*. *maximus* during the annual reproductive cycle. Immunohistochemical staining for BAX and BCL2 (A) and expression of *BAX*, *BCL2* and apoptotic index (AI) measured by real time PCR (B) in adult active, inactivating, inactive and activating testis. ST: seminiferous tubules, SG: spermatogonia; PS: primary spermatocyte; SS: secondary spermatocyte; SP: spermatid. Scale bar: 50 μm. Values indicate mean ± SEM. Different letters indicate significant differences between groups (*p*<0.05).

The expression of two of the most important regulators of the intrinsic pathway of apoptosis, BAX as pro-apoptotic marker and BCL2 as anti-apoptotic marker, was analyzed by immunohistochemistry and real time PCR. Within the seminiferous tubules, BAX immunostaning was observed mainly in spermatocytes in active, inactivating and activating testis and in a few spermatogonia in inactive testis (Fig 4A). On the other hand, BCL2 was mainly localized in spermatocytes and early spermatids in active and activating testis, whereas it was restricted to spermatogonia in inactivating and inactive testis (Fig 4A). Some Leydig and Sertoli cells were also positive for BAX and BCL2 (Fig 4A). No differences were detected in the expression of *BCL2* throughout the annual reproductive cycle while the expression of *BAX* decreased significantly in the activating testis, generating a lower AI (*BAX*/*BCL2* relative expression ratio) in this group compared to the other testicular stages (Fig 4B).

### Autophagy varies in a season-dependent manner in the testis of the male vizcacha

**Fig 5:**
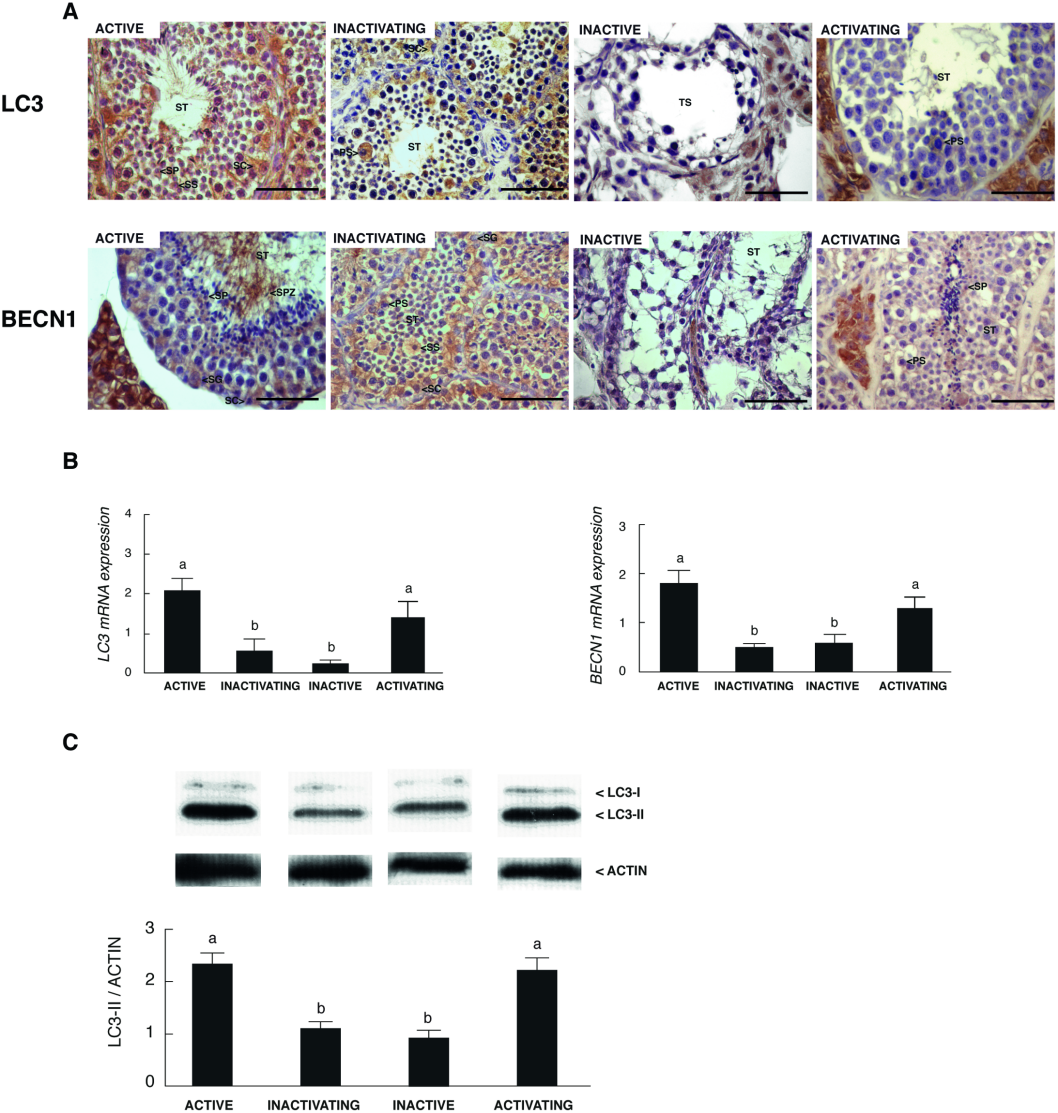
Localization and expression of autophagic- related genes/proteins in the testis of *L*. *maximus* during the annual reproductive cycle. Immunostaning (A) and mRNA expression (B) for BECN1 and LC3 and (C) LC3I/II protein levels in adult active, inactivating, inactive and activating testis. ST: seminiferous tubules, SG: spermatogonia; PS: primary spermatocyte; SS: secondary spermatocyte; SP: spermatid; SPZ: spermatozoid; SC: Sertoli cell. Scale bar: 50 μm. Values indicate mean ± SEM. Different letters indicate significant differences between groups (*p*<0.05).

We analyzed the expression of two autophagy-related genes/proteins: i) BECLIN 1 (BECN1), involved in the formation of the pre-autophagosomal structure, and ii) microtubule-associated protein 1 light chain 3 (LC3) which is cleaved and associated with the autophagosome membrane indicating that autophagy is indeed occurring [15]. BECN1 and LC3 were immunolocalized in the seminiferous tubules, both in the germ line and Sertoli cells, in active, inactivating and activating testes (Fig 5A). In the inactive testis, both BECN1 and LC3 ceased to be immunodetected in the tubular compartment (Fig 5A). The expression of BECN1 and LC3 was also detected in the Leydig cells in all groups analyzed (Fig 5A). The mRNA analysis of both autophagy-related genes showed that the expression on *BECN1* and *LC3* was lower in inactive and inactivating testis respect to the other groups (Fig 5B). Additionally, we decided to evaluate the protein expression of LC3I/II, an indicator of autophagosome formation. Western blot analysis showed a significant increase of LC3II in the active and activating testes respect to the other groups (Fig 5C).

### Nutritional stress regulates the expression of autophagy-related genes in the testis

**Fig 6:**
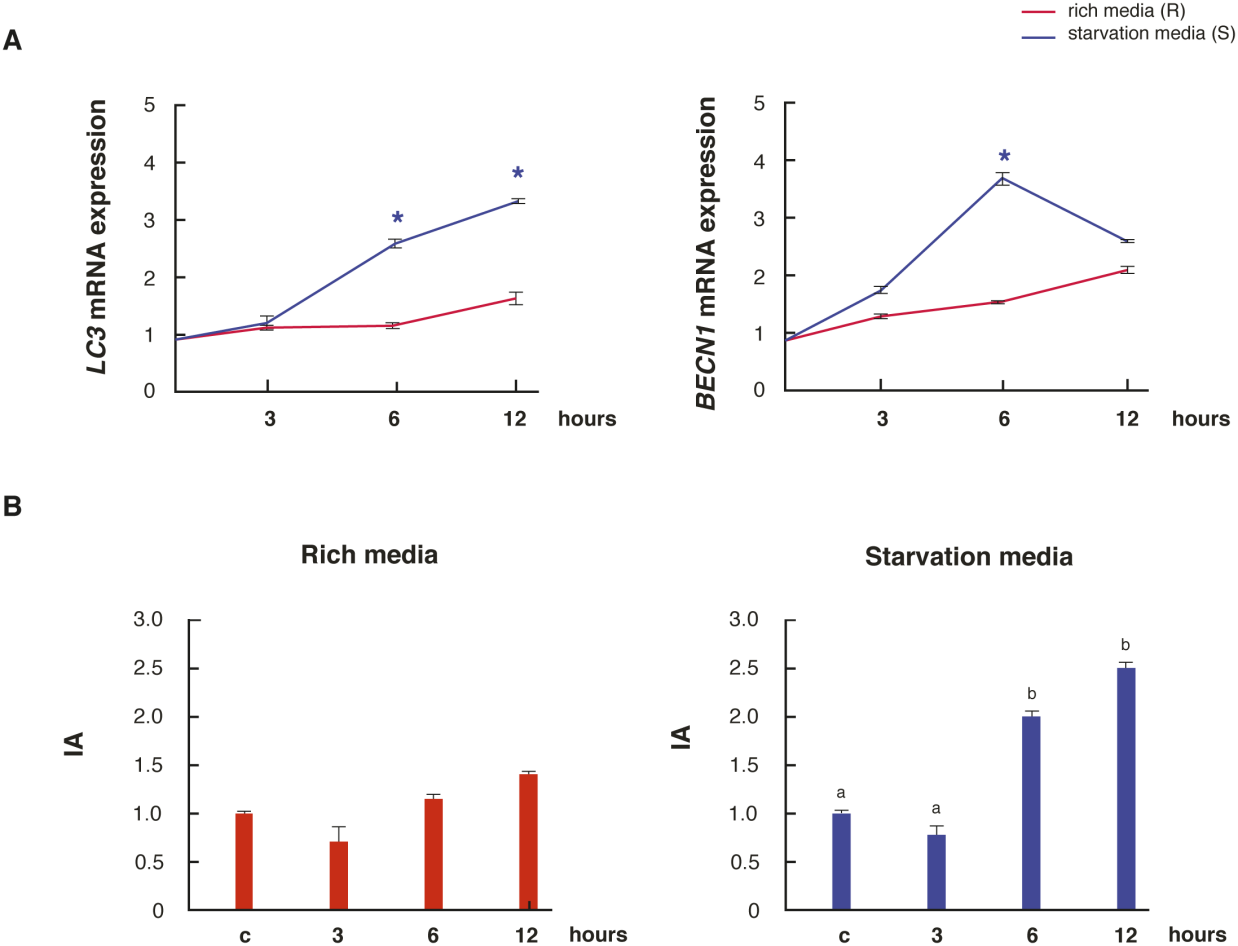
Testicular regulation of autophagic and apoptotic-related genes in nutritional stress conditions. (A) Adult active testes were incubated in rich or starvation media and the mRNA expression of *BECN1* and *LC3* levels were measured. (B) Expression of testicular AI in adult active testes incubated in reach or starvation media. Values are expressed as mean ± SEM. * indicates significant differences between media for each incubation time (*p*<0.05).

Since autophagy is a pathway of survival, cell death or a combination of both, we decided to analyze the expression of the autophagic- and also the apoptotic- markers in active testis explants incubated under normal nutritional conditions (rich media) and nutritional stress (starvation media). Starvation increased the mRNA expression of *BECN1* and *LC3* at 6 h and 6/12 h respectively, compared to rich media (Fig 6A). Concerning apoptosis, we decided to evaluate whether the AI varied over time in the testis explants subjected to the rich and starvation media. The testicular AI remained unchanged over time in rich media, while it increased at 6 and 12 h of incubation in the starvation media respect to control (Fig 6B).

## Discussion

In seasonal breeding animals, the testicular mechanisms that regulate the transition between the active (functional) and inactive (regression) stage vary between species. Variations from one to the other stage were mainly associated to apoptosis or proliferation [2,3,13]. Here, we showed the autophagy as a contributing mechanism to the changes in the seminiferous epithelium in the seasonal breeder basal rodent *L*. *maximus*. Even more, we suggest the existence of a balance between apoptosis and autophagy within the seminiferous tubules of the adult active testis, whose alteration promotes germ cell loss during testis regression.

The regressed testis of *L*. *maximus* showed a marked decrease of the volume of the seminiferous tubules that turned into cords, composed mainly of spermatogonia and Sertoli cells. This was expected for this time-point of the reproductive cycle and it is consistent with observations in other seasonal animals such as the hamster and the white-footed mice [2,30]. The number of proliferating spermatogonia increased gradually from the active to the activating stage of the testis. In this way, a proliferation wave could exist in the months prior to the breeding season, as it has been described in the brown hare [10], the roe deer [31] and the Iberian mole [3]. This data suggest that proliferation imbalances may not be involved in the regression of the seminiferous epithelium of *L*. *maximus*, but could be responsible for reestablishing the spermatogonia pool during the activating stage of the testis.

In several species, apoptosis of male germ cells is the cause and one of the earliest events associated with testicular regression [7,8,10]. However, in other cases, proliferation rather than apoptosis is responsible for testicular germ cell loss [3,13]. Here, we did not observe significant changes in the number of apoptotic spermatogonia and meiotic germ cells from the active to the inactive testicular reproductive stage of *L*. *maximus*. The analysis of markers involved in the intrinsic apoptotic pathway revealed that the localization of BAX and BCL2 was adluminal in the active testes and began to localize in spermatogonia in the posterior reproductive testicular stages of *L*. *maximus*. Interestingly, no apoptotic germ cells were detected in the activating testes; moreover, the AI was the lowest at this point of the reproductive cycle. This is in agreement with the high levels of proliferating spermatogonia detected in the activating stage that decay once the testis returns to be functional. In line with these results, it has been described that apoptosis is also suppressed during the activating phase in the Djungarian hamster [32] and the bullfrog [33]. Since in the active, inactivating and activating testes apoptotic germ cells were always observed, it seems reasonable to consider the participation of alternative mechanisms that explain the massive loss of germ cells in regressed testis. Autophagy has been scarcely studied in male reproduction being the information referred to the testis almost nonexistent. Recently, it has been demonstrated that Sertoli and Leydig cells unable to produce autophagy generates sterile phenotypes due to total loss of germ cells and production of acephalic spermatozoa, respectively [34]. Here, we originally reported the presence of two autophagic regulators, BECN1 and LC3, in germ cells as well as in Leydig and Sertoli cells, which were also regulated during the annual cycle and by nutritional stress. In the adult active and activating testis of *L*. *maximus*, both autophagic regulators were increased in spermatogonia and meiotic germ cells. Moreover, LC3II protein levels were the highest in these two testicular stages. Therefore, we could infer that if autophagy acts as an alternative mechanism of cell death, it would be to eliminate damaged germ cells or the cytoplasmic surplus of spermatids during spermiogenesis, as it was previously described for the mouse [17]. Here, we reported for the first time, a positive regulation of *BECN1* and *LC3* under starvation conditions in the testis. We also observed that testicular apoptosis was induced under nutritional stress conditions. Since the intrinsic pathway of apoptosis can interact with autophagic-related proteins, the balance between these two processes could determine the final destination of the cells. Recently, Liu et al (2016) showed that autophagy pathway, throughout LC3 expression, is involved in the de-differentiation of the seminiferous tubules during the testicular regression in the plateau pika [35]. On the contrary, we observed that when the testis start to regress and the metabolic requirement diminish, the protein expression of LC3I/II and the gene expression of *BECN1* and *LC3* decreased. During testicular recovery, the mRNA and protein expression of BECN1 and LC3 began to increase restoring the balance between apoptosis and autophagy processes in the active testis. This prompts us to propose that the reduction of autophagy during the inactivating and inactive stages may contribute to the loss of germ cells, not as a direct effect of this process, but as a consequence of the absence autophagic-related proteins.

Different stress conditions could trigger autophagy and apoptosis within a single cell. Generally, autophagy blocks the induction of apoptosis, and apoptosis (via caspases induction) may block the autophagic process [15]. However, it is impossible to state whether autophagy would be influencing apoptosis or vice versa, or whether it would be acting as a mechanism of cell death or survival prior to induction of apoptosis in the testis of *L*. *maximus*. The scarce information regarding autophagy makes all these scenarios possible. In conclusion, we propose the existence of a dialogue between apoptosis and autophagy that would be highly dependent on the cellular context and could modulate the changes in the seminiferous epithelium during the reproductive annual cycle of *L*. *maximus*. Therefore, it is important to consider that an imbalance between both processes would have important pathophysiological consequences.
